# Catatonia and jaw dislocation in the postoperative period with epidural morphine

**DOI:** 10.4103/0019-5049.79904

**Published:** 2011

**Authors:** Satyen Parida, Varsha D Allampalli, Sudeep Krishnappa

**Affiliations:** Department of Anaesthesiology and Critical Care, JIPMER, Pondicherry, India

**Keywords:** Catatonia, epidural morphine, post-operative, temporomandibular joint dislocation

## Abstract

We report a case of temporomandibular joint dislocation occurring in the postoperative period in a patient, who developed catatonia following administration of a single dose of epidural morphine. The catatonic response to epidural morphine was delayed by several hours in the postoperative period, and might have resulted from intrathecal migration of the drug, through an initial dural puncture while locating the epidural space. The temporomandibular joint dislocation was diagnosed only after reversal of the effects of morphine with naloxone, when the patient complained of inability to fully close her mouth.

## INTRODUCTION

Epidural morphine has been used for postoperative analgesia. However, many side effects, such as nausea, vomiting, pruritus, urinary retention, delayed respiratory depression, and mental status changes have been reported with its use.

Temporomandibular joint injuries accounted for 10% of airway trauma claims (27 of 266 claims) in the American Society of Anesthesiologists (ASA) Closed Claims database and were associated with routine tracheal intubation in all cases. Most temporomandibular joint (TMJ) claims were submitted by women and by patients younger than 60 years.[1] Sixteen of the claims were for TMJ pain and 11 were for TMJ dislocation.

## CASE REPORT

A 48-year-old, 48 kg, 154-cm tall woman, ASA physical status II, posted for total abdominal hysterectomy and bilateral salpingo-oophorectomy, was scheduled for a combination of continuous lumbar epidural analgesia and general anaesthetic. The surgery was indicated for management of an ovarian tumour. She had a history of some psychiatric illness a few years back and was on medications, which were later discontinued, but details of the same could not be obtained owing to poor documentation. During location of the epidural space, the initial attempt resulted in a dural puncture, following which the epidural space was located at the immediate upper spinous interspace and anaesthesia proceeded uneventfully with endotracheal intubation. She was extubated on table, Inj morphine 3 mg administered through the epidural catheter for relief of postoperative pain, and was shifted to the postoperative ward without any major concerns.

Five hours later, she was found catatonic in bed with total loss of limb movements. Plantars were flexor and pupils normal. Eyes were half-open, mouth slightly open and the patient held this posture ignoring any verbal contact or external stimulus. She was actively resisting eyelid elevation and blinking in response to visual threat. The patient was haemodynamically stable and arterial blood gases (ABG), serum electrolytes, and blood glucose levels were unremarkable. In view of the previous dural puncture, egress of the epidurally administered morphine into the intrathecal space was considered and naloxone administered to antagonize its effects. In about an hour, the patient was fully conscious, oriented, and responding to oral commands. However, the patient was not vocalizing and was still keeping her mouth slightly open. Upon being instructed to close her mouth, the patient indicated that she was unable to do so and TMJ dislocation was considered. The Oral and Maxillofacial Surgeon was called to do a reduction of the TMJ. The patient’s jaw was placed in a bandage for 2 days with instructions to refrain from opening her mouth widely for several weeks and to support her jaw while yawning.

## DISCUSSION

Mental status changes may occur after intrathecal and epidural opioids. While sedation is the most common of such mental status changes, naloxone-reversible catatonia[2,3] has also been reported.

TMJ dislocation occurs when the mandibular condyle prolapses anteriorly over the articular eminence and is completely displaced forward of the articular fossa. Displacement of the articular disc behind the head of the condyle contributes to the obstruction to closing. When dislocation is present, the patient’s mouth is widely open, only the molar teeth are in apposition and the mouth cannot be closed. Yawning or wide opening of the mouth makes the joint vulnerable to dislocation as the ligaments supporting the articular disc are most lax at this time.

We report a case of catatonia developing several hours after administration of a single dose of epidural morphine, and TMJ dislocation, presumably occurring due to abnormal posturing of the mouth during the catatonic response. It is possible that the dural hole resulting from the previous “wet tap” might have resulted in some of the morphine entering the intrathecal space, which could have caused greater concentrations of morphine being delivered to the central neuraxis, evoking such a response in a patient with a history of prior psychosis. The potential for leakage of solution through the dural hole into the subarachnoid space is always there.[4] We did not encounter any evidence, either clinical or biochemical, as reflected in the ABG, of significant respiratory depression. The patient had been observed to be able to close her mouth normally in the immediate postoperative period. Hence we presume that the TMJ dislocation might have occurred in the postoperative ward, possibly due to abnormal posturing of the mouth during the catatonic episode.

The patient was administered tab. metoclopramide 10 mg as part of her premedication on the morning of surgery. Hence, possibility of “Serotonin syndrome” could be considered. The symptoms of such a syndrome are often described as a triad of clinical abnormalities:

- Cognitive effects: Headache, agitation, hypomania, mental confusion, hallucinations, coma
- Autonomic effects: Shivering, sweating, hyperthermia, hypertension, tachycardia, nausea, diarrhoea.
- Somatic effects: Myoclonus (muscle twitching), hyperreflexia (manifested by clonus), tremor.

However, the timing of demonstration of such symptoms in our patient (evening of surgery, nearly 5 hours postoperative) and the symptomatology that she demonstrated, with an absence of any autonomic or somatic signs, argues against a “Serotonin syndrome” - like effect. Moreover, the response to naloxone was also indicative of an “opioid aetiology” than otherwise. No other drugs were administered in the immediate postoperative period, which could have had a similar effect.

In this case, we administered a test dose with lignocaine and epinephrine to quiz the epidural catheter for possible intravascular or subarachnoid placement prior to inducing the patient, and the test was negative. Furthermore, the same catheter was utilized intraoperatively for maintenance of analgesia with small boluses of local anaesthetics and no untoward haemodynamic effects were encountered.

Our patient denied any past episodes of similar dislocation of the TMJ requiring attention. Overuse of masticator muscles, often associated with psychological stress or bruxism is a possible physical cause of instability of the TMJ. Since our patient had a history of previous psychiatric illness, the details of which we failed to unearth, the possibility that she might have had TMJ instability related to her psychological state cannot be discounted.

Cases have been reported in which diagnosis of TMJ dislocation was missed intraoperatively, and could only be made after the patient regained consciousness and complained of preauricular pain and inability to close the mouth.[5,6] In patients with a history of TMJ instability, apart from taking proper history and physical examination, it might be a good idea to check the mandibular occlusion just prior to induction so that it can be compared with the occlusion after airway manipulation in order to diagnose dislocation of the joint even in the unconscious patient.

We cannot outline the mechanisms involved in development of catatonia in our patient, nor are we in a position to clearly explain why such complication was delayed by several hours after initial drug administration. Presumably, mechanisms similar to those involved in delayed respiratory depression after administration of morphine for central neuraxial blockade would explain the time delay. Mental status change caused by intrathecal and epidural opioids likely results from cephalad migration of drug in cerebrospinal fluid and subsequent interaction with opioid receptors located in the brain. Possible mechanisms include interactions with opioid receptors located in the thalamus, limbic system, and cerebral cortex.[7]

In summary, the case demonstrates that TMJ dislocation can not only occur during airway manipulation under anaesthesia, but can also occur in the postoperative period in a sedated or unconscious patient.

## References

[1] Domino KB, Posner KL, Caplan RA, Cheney FW (1999). Airway injury during anaesthesia. A closed claims analysis. Anesthesiology.

[2] Rawal N, Arner S, Gustafsson LL, Allvin R (1987). Present state of extradural and intrathecal opioid analgesia in Sweden. Br J Anaesth.

[3] Engquist A, Chraemmer Jorgensen B, Anderson HB (1980). Epidural morphine-induced catatonia. Lancet.

[4] Leach A, Smith GB (1986). Subarachnoid spread of epidural local anaesthetic following dural puncture. Anaesthesia.

[5] Rastogi NK, Vakharia N, Hung OR (1997). Perioperative anterior dislocation of the temporomandibular joint. Anesth Analg.

[6] Gambling DR, Ross PL (1988). Temporomandibular joint subluxation on induction of anesthesia. Anesth Analg.

[7] Shulman MS, Sandler A, Brebner J (1984). The reversal of epidural morphine induced somnolence with physostigmine. Can Anaesth Soc J.

